# Intrinsic defence capacity and therapeutic potential of natriuretic peptides in pulmonary hypertension associated with lung fibrosis

**DOI:** 10.1111/bph.12694

**Published:** 2014-06-25

**Authors:** R S Baliga, C J Scotton, S L Trinder, R C Chambers, R J MacAllister, A J Hobbs

**Affiliations:** 1 William Harvey Research Institute, Barts & The London School of Medicine, Queen Mary University of LondonCharterhouse Square, London, UK; 2 Centre for Respiratory Research, University College LondonThe Rayne Building, London, UK; 3 Centre for Clinical Pharmacology, University College LondonThe Rayne Building, London, UK

**Keywords:** natriuretic peptide, neutral endopeptidase, guanylyl cyclase, cyclic GMP, pulmonary hypertension, phosphodiesterase, bleomycin

## Abstract

**BACKGROUND AND PURPOSE:**

Idiopathic pulmonary fibrosis (IPF) is a progressive fibro-proliferative disorder refractory to current therapy commonly complicated by the development of pulmonary hypertension (PH); the associated morbidity and mortality are substantial. Natriuretic peptides possess vasodilator and anti-fibrotic actions, and pharmacological augmentation of their bioactivity ameliorates renal and myocardial fibrosis. Here, we investigated whether natriuretic peptides possess an intrinsic cytoprotective function preventing the development of pulmonary fibrosis and associated PH, and whether therapeutics targeting natriuretic peptide signalling demonstrate efficacy in this life-threatening disorder.

**EXPERIMENTAL APPROACH:**

Pulmonary haemodynamics, right ventricular function and markers of lung fibrosis were determined in wild-type (WT) and natriuretic peptide receptor (NPR)-A knockout (KO) mice exposed to bleomycin (1 mg·kg^−1^). Human myofibroblast differentiation was studied *in vitro*.

**KEY RESULTS:**

Exacerbated cardiac, vascular and fibrotic pathology was observed in NPR-A KO animals, compared with WT mice, exposed to bleomycin. Treatment with a drug combination that raised circulating natriuretic peptide levels (ecadotril) and potentiated natriuretic peptide-dependent signalling (sildenafil) reduced indices of disease progression, whether administered prophylactically or to animals with established lung disease. This positive pharmacodynamic effect was diminished in NPR-A KO mice. Atrial natriuretic peptide and sildenafil synergistically reduced TGFβ-induced human myofibroblast differentiation, a key driver of remodelling in IPF patients.

**CONCLUSIONS AND IMPLICATIONS:**

These data highlight an endogenous host-defence capacity of natriuretic peptides in lung fibrosis and PH. A combination of ecadotril and sildenafil reversed the pulmonary haemodynamic aberrations and remodelling that characterize the disease, advocating therapeutic manipulation of natriuretic peptide bioactivity in patients with IPF.

## Introduction

Idiopathic pulmonary fibrosis (IPF) is a progressive and fatal lung disorder resulting from unrelenting matrix deposition and diminution of lung function (Bjoraker *et al*., 1998; Eickelberg and Selman, 2010). IPF is the most common interstitial lung disease (ILD) and carries the worst prognosis, with median survival ranging from 2 to 3 years (Martinez *et al*., 2005; Eickelberg and Selman, 2010; Ley *et al*., 2011). Pulmonary hypertension (PH), characterized by increased pulmonary vascular resistance, remodelling of the small pulmonary arteries and right ventricular hypertrophy (RVH), is a common complication in IPF (affecting 30–40% of patients), and individuals with a dual diagnosis exhibit markedly reduced survival (Lettieri *et al*., 2006; Mejia *et al*., 2009). PH is also frequently associated with other ILDs, including systemic sclerosis-driven pulmonary fibrosis, where it also results in increased mortality (Behr and Ryu, 2008; Condliffe *et al*., 2009). IPF is refractory to virtually all current therapeutic options and the recently licensed anti-fibrotic, anti-inflammatory drug pirfenidone achieves only a small treatment benefit at high financial cost (Noble *et al*., 2011). Therefore, identification of novel mechanisms underpinning pathogenesis and potential therapeutic targets that possess the ability to combat both the altered pulmonary haemodynamics and fibro-proliferative lung phenotype is of high priority.

Natriuretic peptides are a family of vasodilator hormones that play a pivotal role in the regulation of blood volume and blood pressure (Lee and Burnett Jr, 2007; Potter *et al*., 2009). Atrial natriuretic peptide (ANP) and brain natriuretic peptide (BNP) are released predominantly from the heart, while a third member of this family, C-type natriuretic peptide, is released principally from endothelial cells. These peptides act via guanylyl cyclase-linked receptors to promote cGMP production, salt and water excretion, and peripheral vasodilatation. Natriuretic peptides and their cognate receptors have also been localized to many areas of the lung, including peripheral lung tissues, airway epithelium and pulmonary vasculature (Bianchi *et al*., 1985; Sakamoto *et al*., 1986; Gutkowska *et al*., 1987; Toshimori *et al*., 1988; receptor nomenclature follows Alexander *et al*., 2013), and are thought to play a key role in lung physiology, regulating several processes, including vasodilation, bronchorelaxation, pulmonary permeability and surfactant production (Hulks *et al*., 1990; Perreault and Gutkowska, 1995).

We have recently reported that augmentation of endogenous natriuretic peptide bioactivity, using ecadotril, an inhibitor of neutral endopeptidase [NEPi; an enzyme that metabolizes and inactivates natriuretic peptides (Kenny and Stephenson, 1988; Soleilhac *et al*., 1992)] in tandem with sildenafil, an inhibitor of phosphodiesterase 5 [PDE5i, hydrolyses and inactivates cGMP (Bender and Beavo, 2006)] and first-line treatment of PH, prevents and reverses pathogenesis in a hypoxic model of PH (Baliga *et al*., 2008). The natriuretic peptide family is also known to exert anti-proliferative and anti-fibrotic bioactivity in the heart and kidney (Tamura *et al*., 2000; Knowles *et al*., 2001). Here, we have used a well-characterized model of lung fibrosis induced by bleomycin, to establish a role for endogenous natriuretic peptides in curbing pathogenesis, and the therapeutic potential of this combination of NEPi PDEi in reversing the haemodynamic dysfunction and fibrotic processes that underpin lung fibrosis.

## Materials and methods

### Bleomycin-induced lung fibrosis

All animal care and experimental studies conformed to the UK Animals (Scientific Procedures) Act 1986 and were approved by the appropriate ethical committee of Queen Mary University of London. All studies involving animals are reported in accordance with the ARRIVE guidelines for reporting experiments involving animals (Kilkenny *et al*., 2010; McGrath *et al*., 2010). A total of 160 animals were used in the experiments described here.

Mice were housed in a pathogen-free facility with access to food and water *ad libitum*. Littermate wild-type (WT) and natriuretic peptide receptor (NPR)-A knockout (KO) mice (C57B6; male; 20–25 g; kind gift of O. Smithies, University of North Carolina) were exposed to bleomycin (50 μL·mouse^−1^; 1 mg·kg^−1^ = ∼25 IU·per mouse) by oropharyngeal instillation under light isofluorane anaesthesia. Controls were similarly instilled with 50 μL of sterile saline. Animals were randomly assigned to the following groups:

- Control: saline-treated and receiving daily gavage with vehicle for ecadotril (0.3 mL). This vehicle consists of 0.1% polyethylene glycol + 0.5% carboxymethyl cellulose in water.
- Bleomycin: bleomycin-treated and receiving daily gavage with vehicle
- Bleomycin + sildenafil: bleomycin-treated and receiving sildenafil (30 mg·kg^−1^ in drinking water) and daily gavage of vehicle
- Bleomycin + ecadotril: bleomycin-treated and receiving daily gavage of ecadotril (60 mg·kg^−1^ in vehicle)
- Bleomycin + sildenafil + ecadotril: bleomycin-treated and receiving sildenafil (30 mg·kg^−1^ in drinking water) plus daily gavage of ecadotril (60 mg·kg^−1^ in vehicle)

Treatment was started concomitantly with the administration of bleomycin (i.e. day 0) and outcome measures assessed at day 14. Doses of sildenafil and ecadotril were chosen based on previous work (Stasch *et al*., 1995; Zhao *et al*., 2001; Baliga *et al*., 2008).

In further experiments, to determine the ability of natriuretic peptides to reverse existing pulmonary fibrosis, mice were exposed to bleomycin (as above), with treatment initiated at day 14 and analysis undertaken at day 28. This ‘therapeutic dosing schedule’ was chosen after thorough, temporal evaluation of the pathogenesis of the bleomycin model in our laboratory (Scotton and Chambers, 2010), ensuring the progression of established fibrosis was targeted.

### *In vivo* haemodynamic and morphological analysis

Animals were anaesthetized using isofluorane (1.5%, 0.2 mL·min^−1^ oxygen), and right ventricular systolic pressure (RVSP) and mean arterial blood pressure (MABP) were determined as described previously (Baliga *et al*., 2008). Following haemodynamic measurements, animals were killed by exsanguination (under isofluorane anaesthesia, as above), hearts excised, and right ventricle to left ventricle plus septum [RV/(LV + S)] ratio determined as a measure of RVH.

### Tissue processing and histochemistry

Inflated lungs were fixed in 4% paraformaldehyde for 4 h at 4°C and then incubated overnight in 15% sucrose in PBS at 4°C. Tissue was dehydrated in 70% ethanol and embedded in paraffin. Serial sections (4 μm) were used for MSB (Martius/Scarlet/Blue; DAKO, Ely, UK) staining and α-smooth muscle actin (αSMA) immunohistochemistry. For the latter, sections were incubated with a mouse monoclonal anti-αSMA antibody (DAKO; 1:1000 dilution), followed by biotinylated anti-mouse secondary antibody. Immunoreactivity was detected using the ABC-peroxide based system (DAKO) following the manufacturer's protocol. Stained slides were imaged by Nanozoomer virtual microscopy (Hamamatsu, Welwyn Garden City, UK). Pulmonary arterial muscularization was then assessed as previously described (Baliga *et al*., 2008). Briefly, vessels were defined according to the presence or absence of αSMA positive staining. Twenty five muscularized arteries from different fields were imaged at 400 × magnification by light microscopy from representative animals in each group to determine wall thickness. All histology slides were coded and evaluated without knowledge of the treatments.

### Total lung collagen analysis

Collagen content was determined by measuring hydroxyproline by reverse-phase HPLC of 7-chloro-4-nitrobenzo-oxao-1,3,-diazole-derived acid hydrolysates; the total lung collagen was then calculated in mg, assuming lung collagen contains 12.2% (w/w) hydroxyproline (Scotton *et al*., 2009).

### Human fibroblast differentiation

The ability of ANP (1 μM) and sildenafil (3 μM) alone, or in combination, to prevent TGFβ-driven (1 ng·mL^−1^) human lung fibroblast differentiation was evaluated as we have described previously using primary human lung fibroblasts isolated from macroscopically healthy segments of lung from patients undergoing lung cancer resection (kind gift of Dr. R.J. McAnulty, University College London) (Scotton *et al*., 2009).

### Quantitative real-time polymerase chain reaction for pro-proliferative markers

The expression of mRNA for several pro-proliferative biomarkers was conducted as we have described previously (Scotton *et al*., 2009). Primers for inducible nitric oxide synthase (iNOS), collagen (Col)-1a and β-actin were from Primer Design (Southampton, UK); the others are shown in Table 1.

**Table 1:** Primer sequences (5′→3′) used to quantify lung mRNA expression

GENE	Primer sequence
αSMA	Forward: AGAGTGGAGAAGCCCAGCCAGT
	Reverse: CCAGAGCCATTGTCGCACACCA
TGFβ	Forward: GGATACCAACTATTGCTTCAGCTCC
	Reverse: AGGCTCCAAATATAGGGGCAGGGTC
IL-1β	Forward: GACCTTCCAGGATGAGGACA
	Reverse: CTAATGGGAACGTCACACACC
IL-13	Forward: CCTGGCTCTTGCTTGCCTT
	Reverse: GGTCTTGTGTGATGTTTGCTCA
TNFα	Forward: CAAATGGCCTCCCTCTCAT
	Reverse: CACTTGGTGGTTTGCTACGA

### Biochemical analyses

Plasma ANP (Phoenix Pharmaceuticals, Karlsruhe, Germany) and cGMP (GE Healthcare, Hatfield, UK) concentrations were measured using commercially available ELISA.

### Data analysis

Results are expressed as means ± SEM. Data were analysed by one-way ANOVA with a Bonferroni post test where appropriate. A Shapiro–Wilk test was applied to data sets to confirm normal distribution. *P* < 0.05 denotes significance.

### Materials

Bleomycin was supplied by Kyowa Hakko Kirin Co. Ltd, Tokyo, Japan; ecadotril was a kind gift of Dr. Johannes-Peter Stasch, Bayer AG, Wuppertal, Germany. TGFβ was from R&D Systems, Oxford, UK; ANP was supplied by Cambridge Bioscience, Cambridge, UK; isofluorane was from Abbott Laboratories, Maidenhead, UK. Sildenafil was extracted from tablets (Viagra, Pfizer, UK; dispensed by the UCLH pharmacy) by crushing and dissolving in warm (40^o^C) water, filtration and then acidification with HCl. Extraction was achieved with ethyl acetate to give the free base. The citrate was regenerated from the free base by addition of one equivalent of citric acid and warming in water followed by freeze drying. The drinking water containing sildenafil was replaced with fresh solution every 48 hours.

## Results

### Effect of sildenafil and ecadotril on haemodynamic and cardiac parameters

**Figure 1:**
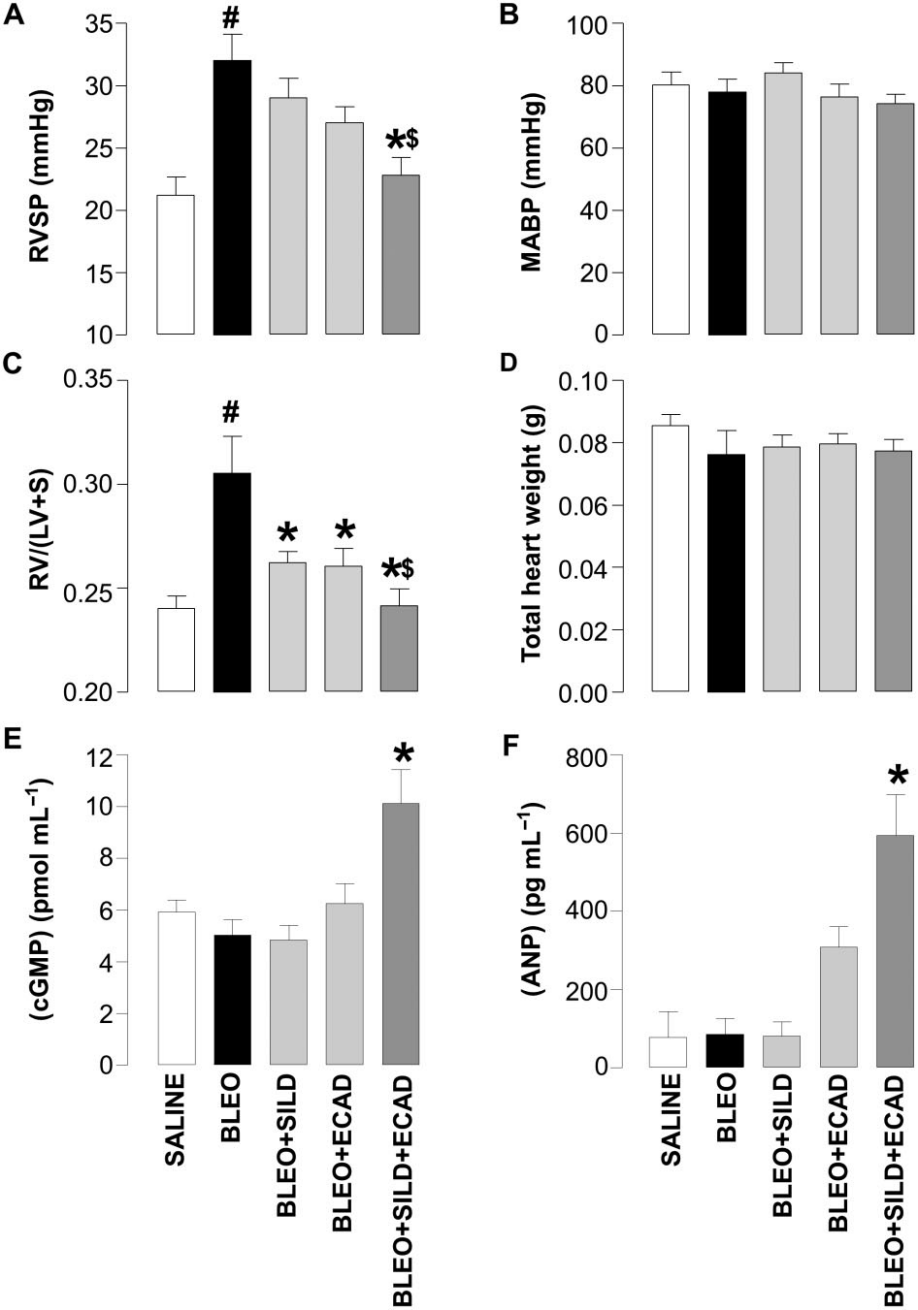
Right ventricular systolic pressure (RVSP) (A), mean arterial blood pressure (MABP) (B), right ventricle : left ventricle plus septum ratio [RV/(LV + S)] (C), total heart weights (D), plasma (cGMP) (E), and plasma (ANP) (F) in saline-treated (control) mice and animals 14 days after exposure to bleomycin (BLEO; 1 mg·kg^−1^) in the absence and presence of sildenafil (SILD; 30 mg·kg^−1^·day^−1^), ecadotril (ECAD; 60 mg·kg^−1^·day^−1^) or sildenafil plus ecadotril (doses as above). ^#^*P* < 0.05 versus saline control; *P < 0.05 versus bleomycin; ^$^*P* < 0.05 versus bleomycin in the presence of sildenafil or ecadotril monotherapy. *n* = 17–25 animals in each group for the haemodynamic measurements, *n* = 3–6 animals for the cGMP and ANP concentrations.

Instillation of bleomycin markedly elevated RVSP compared with saline-treated controls (32.0 ± 5.9 mmHg vs. 21.2 ± 5.9 mmHg; *P* < 0.05). As monotherapy, neither sildenafil nor ecadotril produced a significant reduction in RVSP in mice receiving bleomycin (Figure 1A). However, the combination of sildenafil plus ecadotril caused a significant fall in RVSP when compared with bleomycin-treated mice. Indeed, the effect of combination therapy was so substantial it virtually reduced RVSP to control (saline-treated) levels (∼20 mmHg). Neither sildenafil nor ecadotril, either alone or in combination, caused a significant change in MABP (Figure 1B).

Bleomycin-treated animals showed a significant increase in RV/(LV + S) ratio, confirming the development of RVH in this model. Treatment with sildenafil or ecadotril alone significantly reduced the bleomycin-induced RVH. However, combination treatment with sildenafil plus ecadotril caused an additional reduction in RVH (Figure 1C). There were no significant changes in total heart weight (Figure 1D).

Changes in pulmonary haemodynamics were mirrored by the concentrations of cGMP and ANP in the plasma, which were only significantly increased in the presence of combination therapy (Figure 1E and F).

### Effect of sildenafil and ecadotril on pulmonary vascular remodelling

**Figure 2:**
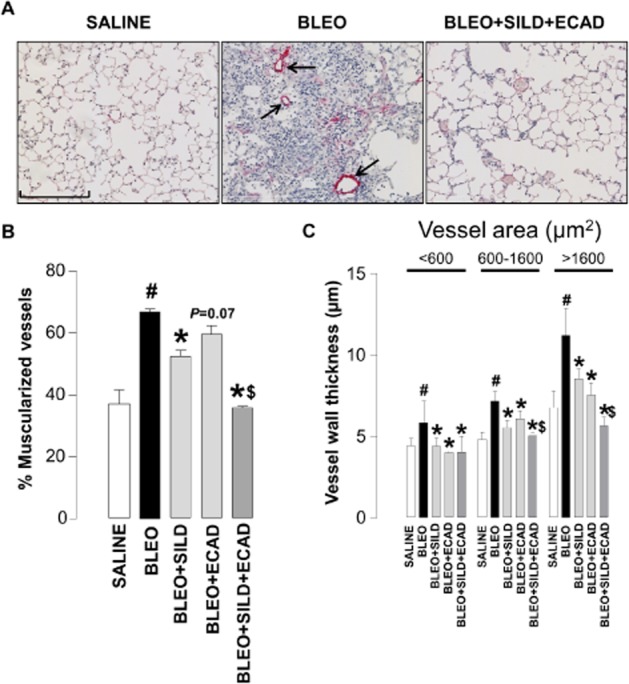
Representative light microscopic images (scale bar = 200 μm) (A), and quantitative assessment of muscularization (B) and wall thickness (C) of pulmonary arteries from saline controls, bleomycin (BLEO; 1 mg·kg^−1^)-treated animals, and mice receiving bleomycin in the presence of sildenafil (SILD; 30 mg·kg^−1^·day^−1^), ecadotril (ECAD; 60 mg·kg^−1^·da^−1^) or sildenafil plus ecadotril (doses as above). Arrows indicate areas of overt muscularization (αSMA staining). ^#^*P* < 0.05 versus saline control; *P < 0.05 versus bleomycin; ^$^*P* < 0.05 versus bleomycin in the presence of sildenafil or ecadotril monotherapy. *n* = 17–25 animals in each group.

Control animals showed only a modest degree of pulmonary muscularization, which was significantly increased by bleomycin (Figure 2A). Treatment with sildenafil, but not ecadotril (*P* = 0.07 vs. bleomycin), caused significant attenuation of this bleomycin-induced muscularization. However, the combination of sildenafil plus ecadotril produced a significantly larger decrease in the percentage of muscularized arteries compared with either treatment alone (Figure 2B). An essentially identical pattern of activity was observed with respect to vessel wall thickness (difference between the internal and external diameter of αSMA-stained vessels (Figure 2C), although in this instance ecadotril alone produced a significant salutary effect on wall thickness.

### Effect of sildenafil and ecadotril on lung fibrosis

**Figure 3:**
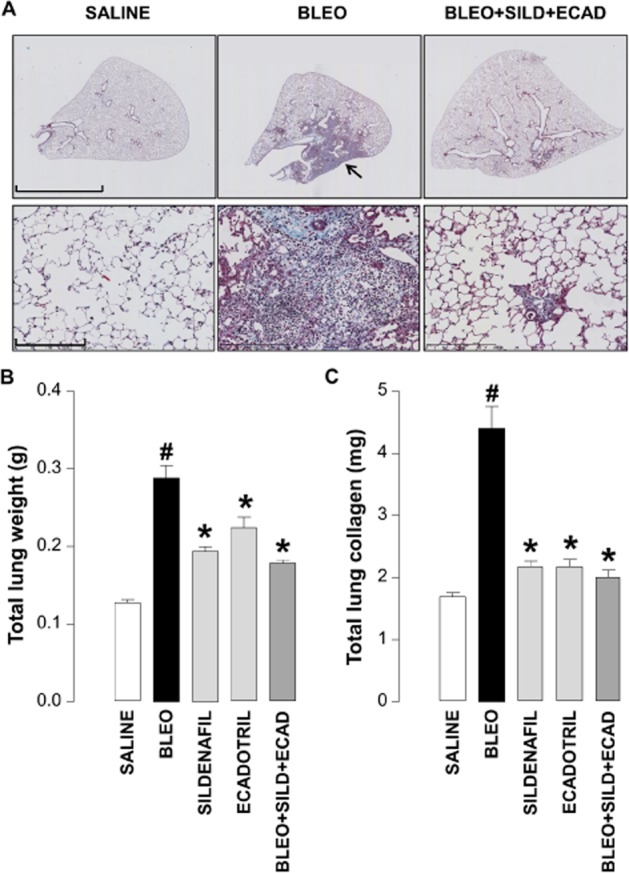
Representative light microscopic images of whole lung cross-sections (upper panels; scale bar = 4 mm) and higher magnification of the same samples (lower panels; scale bar = 200 μm) stained with Martius/Scarlet/Blue (MSB) (A), and quantitative assessment of lung weight (B) and total lung collagen (C) in saline controls, bleomycin (BLEO; 1 mg·kg^−1^)-treated animals, and mice receiving bleomycin in the absence and presence of sildenafil (SILD; 30 mg·kg^−1^·day^−1^), ecadotril (ECAD; 60 mg·kg^−1^·day^−1^) or sildenafil plus ecadotril (doses as above). Arrows indicate areas of overt fibrosis. ^#^*P* < 0.05 versus saline control; *P < 0.05 versus bleomycin. *n* = 17–25 animals in each group.

Bleomycin-injured animals exhibited typical fibrotic lesions consisting of increased cellularity (including fibroblasts) and deposition of extracellular matrix. Mice receiving combination treatment following bleomycin administration showed a clear reduction in the incidence of fibrotic damage (Figure 3A).

Bleomycin administration caused a significant increase in total lung weight (Figure 3B), which was reduced in the presence of sildenafil, ecadotril or the combination (Figure 3B). In accord, bleomycin induced a marked increase in the total lung collagen content that was also attenuated by monotherapy or dual therapy (Figure 3C). In this case, the significant salutary effects of sildenafil and ecadotril *per se* entailed any supplementary advantage with combination treatment was negligible. Nonetheless, the effect of each treatment on collagen content was remarkable, with >80% attenuation in each case.

### Effect of sildenafil and ecadotril on the expression of pro-fibrotic, pro-inflammatory markers in the lung

To provide additional evidence that the combination therapy exerted an anti-fibrotic effect in this model, we determined the expression of mediators known to play a role in the pathogenesis of IPF, including TGFβ, IL-1β, IL-13 and iNOS. In addition, we assessed expression of mRNA for Col1a to confirm the anti-fibrotic activity of combination therapy observed *in vivo* affected collagen production at a transcriptional level.

**Figure 4:**
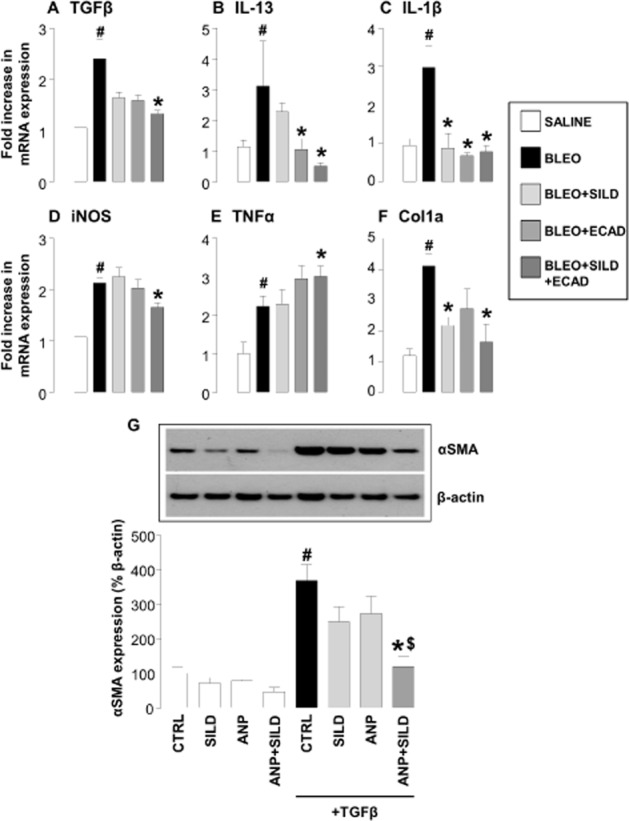
(A–F) Expression of mRNA encoding classical pro-fibrotic/pro-inflammatory mediators in the lungs of saline controls, bleomycin (BLEO; 1 mg·kg^−1^)-treated animals, and mice receiving bleomycin in the presence of sildenafil (SILD; 30 mg·kg·^−1^day^−1^), ecadotril (ECAD; 60 mg·kg^−1^·day^−1^) or sildenafil plus ecadotril (doses as above). ^#^*P* < 0.05 versus saline control; *P < 0.05 versus bleomycin. *n* = 9 experiments for each group. (G) Expression of α smooth muscle actin (αSMA) by human fibroblasts under control conditions or stimulated with TGFβ (1 ng·mL^−1^) in the absence and presence of sildenafil (3 μM), atrial natriuretic peptide (ANP; 1 μM) or sildenafil plus ANP (at the same concentrations). ^#^*P* < 0.05 versus saline control; *P < 0.05 versus TGFβ; ^$^*P* < 0.05 versus TGFβ in the presence of sildenafil or ecadotril monotherapy. *n* = 9 experiments for each group.

Bleomycin treatment resulted in a significant increase in the expression of these mediators, which in accord with effects on collagen deposition was significantly reduced by combination treatment. Monotherapy did reduce the mRNA expression for some mediators (e.g. ecadotril lowered the expression of IL-13), but neither sildenafil nor ecadotril alone was able to produce the wide-ranging inhibitory effect on pro-fibrotic/pro-inflammatory pathways that dual therapy delivered (Figure 4A–D and F). Intriguingly, the expression of TNFα was enhanced by the combination treatment (Figure 4E).

### Effect of sildenafil and natriuretic peptides on human fibroblast differentiation *in vitro*

One of the key steps in the development of pulmonary fibrosis is the differentiation of fibroblasts into αSMA-expressing myofibroblasts (driven principally by TGFβ) that secrete a number of pro-proliferative factors, and are the major source of collagen and extracellular matrix (Burgess *et al*., 2005; Scotton and Chambers, 2007) that precipitate remodelling. To demonstrate that combination therapy was effective in inhibiting this pivotal step in the pathogenesis of fibrosis, and to provide a rudimentary proof of concept in human cells, we explored the ability of natriuretic peptides and PDE5i to block human fibroblast differentiation *in vitro*.

TGFβ caused a rapid differentiation of human lung fibroblasts into myofibroblasts as quantified by the appearance of αSMA positive cells (Figure 4G). In the presence of ANP or sildenafil alone, there was a subtle decrease in the degree of αSMA accumulation. However, the combination of ANP pus sildenafil caused a synergistic inhibition of αSMA up-regulation and fibroblast–myofibroblast differentiation (Figure 4G).

### Effect of NPR-A gene deletion on the development of PH and fibrosis

**Figure 5:**
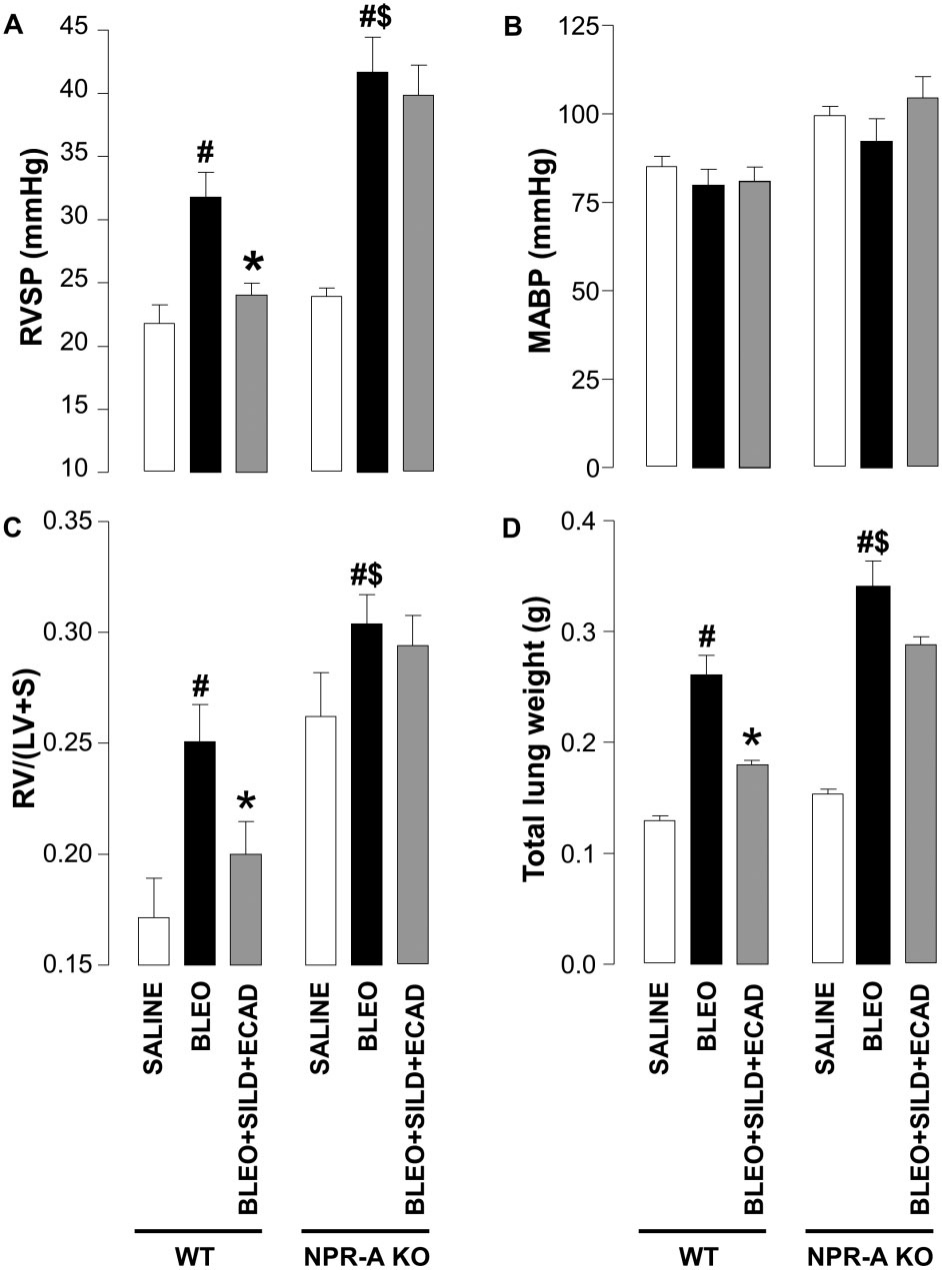
Right ventricular systolic pressure (RVSP) (A), mean arterial blood pressure (MABP) (B), right ventricle : left ventricle plus septum ratio [RV/(LV + S)] (C), and total lung weight (D) in wild-type (WT) mice and natriuretic peptide receptor (NPR)-A KO animals exposed to saline, or bleomycin (BLEO; 1 mg·kg^−1^) in the absence and presence of sildenafil (SILD; 30 mg·kg^−1^·day^−1^) plus ecadotril (ECAD; 60 mg·kg^−1^·day^−1^). ^#^*P* < 0.05 versus saline control; *P < 0.05 versus bleomycin; ^$^*P* < 0.05 versus bleomycin-treated WT. *n* = 6–12 animals in each group.

In order to establish a role for endogenous natriuretic peptides in limiting pathogenesis in IPF, and to confirm that the beneficial effects of combination therapy are dependent on natriuretic peptide bioactivity, we repeated representative studies in NPR-A KO animals. These mice lack the guanylyl cyclase-linked receptor that acts as the principal target for ANP and BNP (Potter *et al*., 2009). In these animals, administration of bleomycin dramatically increased RVSP beyond that observed in WT animals (Figure 5A), suggesting that endogenous natriuretic peptides act in an intrinsic defence capacity. While NPR-A KO animals exhibited a higher MABP, this was not significantly altered by sildenafil, ecadotril or the combination in WT or NPR-A KO mice (Figure 5B). Bleomycin also aggravated RVH in NPR-A KO animals, although this is masked somewhat by the inherent left heart hypertrophy characteristic of this KO strain. Importantly, the beneficial effects of the combination therapy (sildenafil plus ecadotril) were diminished in NPR-A KO mice (Figure 5C).

A similar profile of activity was observed with respect to lung fibrosis. Bleomycin caused an increase in total lung weight that was greater than that reached in WT animals (Figure 5D). Nonetheless, the effect of combination treatment to block the increase in lung weight was blunted in the NPR-A KO mice (Figure 5D).

### Effect of sildenafil and ecadotril on established PH and lung disease

**Figure 6:**
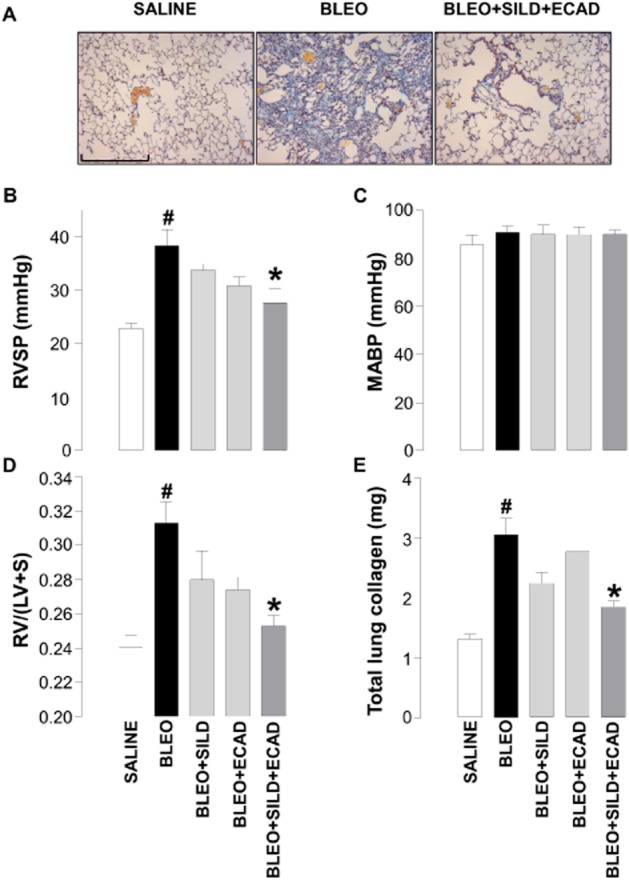
Representative light microscopic images of (scale bar = 200 μm) (A), right ventricular systolic pressure (RVSP) (B), mean arterial blood pressure (MABP) (C), right ventricle : left ventricle plus septum ratio [RV/(LV + S)] (D) and total lung weight (E) in saline controls, bleomycin (BLEO; 1 mg·kg^−1^)-treated animals, and mice receiving bleomycin in the presence of sildenafil (SILD; 30 mg·kg^−1^·day^−1^) plus ecadotril (ECAD; 60 mg·kg^−1^·day^−1^). Sildenafil and ecadotril were administered 2 weeks post-bleomycin exposure. ^#^*P* < 0.05 versus saline control; *P < 0.05 versus bleomycin. *n* = 6–10 animals in each group.

To more closely parallel the clinical situation, additional investigations were conducted in which combination therapy was initiated 2 weeks post-bleomycin, a so-called therapeutic dosing regimen (Scotton and Chambers, 2010). In this case, bleomycin caused the expected increase in RVSP, RVH, total lung collagen and fibrosis (Figure 6A, B, D and E). Despite the delayed administration of treatment, the combination of sildenafil and ecadotril remained effective in reducing each of these parameters (Figure 6A, B, D and E). As with prophylactic treatment, neither sildenafil nor ecadotril, alone or in combination, caused a change in MABP (Figure 6C).

## Discussion

IPF is a progressive fibro-proliferative disorder with a poor prognosis; this is largely the result of a complex and undefined aetiology, and a lack of therapeutic options (Bjoraker *et al*., 1998; Eickelberg and Selman, 2010). ILDs, including IPF, are frequently complicated by the existence of PH; a dual diagnosis that has a far less favourable outcome (Lettieri *et al*., 2006; Mejia *et al*., 2009). We reported recently that augmentation of natriuretic peptide bioactivity results in a significant alleviation of disease severity in hypoxia-induced PH (Baliga *et al*., 2008). This precedent, coupled with the well-defined anti-fibrotic actions of natriuretic peptides in the heart and kidney (Tamura *et al*., 2000; Soeki *et al*., 2005; Li *et al*., 2008; Nishikimi *et al*., 2009; Das *et al*., 2010), provided the rationale to explore the potential of manipulating natriuretic peptide bioactivity in IPF. Data presented here suggest that this approach may offer a substantial pharmacodynamic benefit in IPF and associated PH as it combats both the haemodynamic aberrations and fibro-proliferative aspects of the disease.

Adopting a well-validated model of inflammation and lung injury that is accepted as a model of human lung fibrosis (Scotton and Chambers, 2010), we demonstrate that focusing efforts to maximize cGMP-dependent signalling, using PDE5i and NEPi, offers a potent means of preventing and reversing fibrosis and the accompanying PH. Administration of bleomycin caused the expected increase in lung fibrosis, significantly elevated RVSP and promoted RVH. Neither of the components of the combination therapy (i.e. sildenafil or ecadotril) significantly reduced RVSP at the doses employed. However, this provided the ideal background to reveal a clear synergy between the two drugs when administered together; this cooperative activity mirrored that reported in models of hypoxia-induced PH and patients with the disease (Zhao *et al*., 2003; Preston *et al*., 2004; Klinger *et al*., 2006; Baliga *et al*., 2008). A salutary effect on the right heart was also evident. Interestingly, the effects of the combination therapy were greater (individually and in concert) against structural changes in the right heart compared with the haemodynamic dysfunction. This disparity suggests that augmentation of natriuretic peptide function has direct effects to slow or prevent cardiac hypertrophy, rather than simply secondary to reducing pressure in the pulmonary circulation. This is perhaps not surprising, since all three of the principal members of the natriuretic peptide family exert potent anti-hypertrophic effects in the heart (Tamura *et al*., 2000; Knowles *et al*., 2001). This is a welcome finding since the right heart is often neglected in the consideration of novel therapies for IPF and PH, and treatment modalities that directly preserve right heart structure and function are likely to provide a valuable addition to the therapeutic repertoire. A further key facet of this PDE5i plus NEPi combination is that it appears to target the lung, as MABP was not significantly affected. This selectivity is advantageous in IPF and PH since it avoids the issue of systemic hypotension while being able to maximize cGMP signalling in the pulmonary circulation.

In response to increased resistance, the pulmonary circulation remodels to accommodate the higher pressure and maintain oxygenation. Induction of fibrosis with bleomycin also caused a dramatic increase in the number of muscularized small pulmonary arteries. Akin to the positive effect recorded in hypoxia-induced pH (Baliga *et al*., 2008), here the remodelling of the pulmonary circulation was significantly reduced by monotherapy with PDE5i or NEPi, with a clear additive if not synergistic activity of dual therapy. This provides further evidence that augmenting natriuretic peptide bioactivity exerts a multifaceted beneficial effect on many haemodynamic aspects of pathogenesis in PH linked to IPF. Undoubtedly, if these natriuretic peptide-driven salutary actions translate to the clinical arena, combination treatment should significantly improve outcome. Indeed, the beneficial pharmacodynamic profile of PDE5i plus NEPi holds promise in the treatment of many forms of PH as it appears that the combination therapy is effective in models of PH with contrasting aetiologies.

Combination therapy also produced an impressive reversal of the fibrotic aspects of lung disease. The outcome of prophylactic treatment was manifested as a marked reduction in the total lung weight, collagen deposition, and diminished expression of a number of classically pro-inflammatory and pro-fibrotic genes (e.g. TGFβ, IL-1β, IL-13) (Maher *et al*., 2007; Scotton and Chambers, 2010; Farkas *et al*., 2011) in animals exposed to bleomycin. A major criticism of the bleomycin model is that prophylactic treatment regimens may dampen the inflammatory phase of the response to bleomycin, with a consequent reduction in fibrogenesis (rather than having direct anti-fibrotic efficacy). To address this issue, we also adopted a ‘therapeutic dosing strategy’, administering therapy once fibrosis was already well established. Our data again showed a remarkable effect of the combination therapy on the increase in RVSP, RVH and total lung weight. Facilitation of cGMP-dependent signalling by combination therapy, therefore, exerts a key restraint on one of the central processes leading to overt pulmonary fibrosis, that of extracellular matrix deposition.

To elucidate the mechanism of this anti-fibrotic action, we investigated the effect of natriuretic peptides on fibroblast differentiation (into highly synthetic and contractile αSMA-expressing myofibroblasts), which underpins the synthesis and deposition of extracellular matrix and is perceived to be a key step in the development of IPF (Burgess *et al*., 2005; Scotton and Chambers, 2007). In our experiments, ANP and sildenafil showed no significant effect on fibroblast differentiation individually, but in combination almost completely prevented the differentiation. This fits well with the observations that circulating natriuretic peptide levels increase in patients with pulmonary fibrosis (Burghuber *et al*., 1988; Leuchte *et al*., 2004), advocating the belief that these traditionally cardioprotective mediators offer an innate defence mechanism against (pulmonary) fibrosis. Indeed, there is considerable precedent to support the thesis that natriuretic peptides might exert a wide spectrum of activities that counteract pulmonary fibrosis, thereby enhancing their credentials as a novel therapeutic strategy. For example, natriuretic peptides can prevent TGFβ-induced myofibroblast formation from cardiac fibroblasts, probably by disrupting TGFβ-induced nuclear translocation and downstream signalling of pSmad3 (Kapoun *et al*., 2004). Moreover, in patients with heart failure, TGFβ concentrations inversely correlate with NT-proBNP levels (Behnes *et al*., 2011), and NPR-A KO mice develop renal fibrosis that is associated with increased epithelial–mesenchymal transition and expression of TGFβ (Das *et al*., 2010). Indeed, blockade of Smad signalling appears a common theme in the anti-TGFβ effects of natriuretic peptides since this phenomenon is observed in cardiac fibroblasts, proximal tubular cells and pulmonary artery smooth muscle cells (Kapoun *et al*., 2004; Li *et al*., 2008; Lo *et al*., 2008); whether a similar intervention underlies the beneficial effects of combination therapy in pulmonary fibrosis warrants further attention.

Inhibition of the expression and activity of principal pro-inflammatory and pro-fibrotic cytokines critical in the development of pulmonary fibrosis (Gasse *et al*., 2007; Scotton and Chambers, 2007; 2010) appears another weapon in the anti-fibrotic arsenal of natriuretic peptides. For example, TGFβ is perhaps the best characterized and fundamental driver of fibrotic lung disease; it promotes epithelial activation and dysregulation, and facilitates fibroblast differentiation and proliferation leading to collagen production and deposition (Border and Noble, 1994; Willis and Borok, 2007). The Th_2_ cytokine IL-13 has been shown to promote fibrosis in a number of experimental models (Wynn, 2003), and IL-13-deficient mice exhibit exacerbated lung fibrosis (Kolodsick *et al*., 2004). Inducible NOS also contributes to the development of pulmonary fibrosis and underlies the response to a number of pro-fibrotic cytokines, including IL-5 and IL-13 (Naura *et al*., 2010). iNOS expression is easily identifiable in the lungs of patients with IPF (Saleh *et al*., 1997) and is thought to expedite the development of fibrosis (Naura *et al*., 2010). Each of these classically pro-fibrotic genes showed enhanced expression following bleomycin treatment that was significantly attenuated in mice treated with sildenafil plus ecadotril. This widespread inhibition of the expression of pro-fibrotic genes dovetails well with the favourable effects of combination therapy on the haemodynamic and fibrotic complications of bleomycin exposure, and suggest that pharmacological augmentation of cGMP signalling has a multi-pronged treatment effect. The mechanism(s) underpinning this broad anti-fibrotic/anti-inflammatory influence of combination therapy warrant further attention, but a similar pattern of activity of natriuretic peptides has been reported in acute lung injury (Koga *et al*., 2010); one plausible explanation and common pathway is a cGMP-mediated inhibition of NF-κB activity, perhaps via inhibition of phosphorylation and degradation of the inhibitory subunit IκB, which has been reported *in vitro* and *in vivo* (Moriyama *et al*., 2006; Ladetzki-Baehs *et al*., 2007; Das *et al*., 2010). TNFα levels were also increased in response to bleomycin, consistent with the belief that this cytokine is involved in the pathogenesis of fibrosis (Piguet *et al*., 1993; Zhang *et al*., 1993). Intriguingly, we observed a slight enhancement of TNFα mRNA levels in the bleomycin-treated mice that had received combination therapy. As it was primarily considered a mitogen, anti-TNFα therapies have been evaluated in a variety of fibrotic disorders, but evidence suggests that these approaches may exacerbate disease severity, notably in pulmonary fibrosis (Thavarajah *et al*., 2009). Our data suggest that TNFα has a net anti-fibrotic effect, at least in the bleomycin experimental model, perhaps due to a pro-apoptotic effect via the TNF receptor-1 (Sun and Fink, 2007).

In the present study, by utilizing NPR-A KO mice, we demonstrate that not only does augmentation of natriuretic peptide signalling represent a tangible means to reduce disease severity, but also that endogenous release of natriuretic peptide represents an intrinsic defence mechanism that offsets the progression of pulmonary fibrosis. Using haemodynamic (e.g. RVSP) and structural (e.g. RVH, total lung weight) indices of pathogenesis, in animals lacking innate natriuretic peptide bioactivity (i.e. NPR-A KO) pathology is exacerbated. In addition, the beneficial effects of PDE5i plus NEPi dual therapy were largely absent in NPR-A KO mice. This finding confirms that the principal mechanism that the dual therapy triggers to prevent or reverse pulmonary fibrosis is augmentation of the bioactivity of ANP and/or BNP. This is a key point since NEP metabolizes a number of vasoactive peptides, such as endothelin-1, bradykinin, and vasoactive intestinal peptide (Campbell, 2003), yet it appears that it is primarily the enhancement of the cytoprotective effects of natriuretic peptides that underpins the efficacy of combination treatment in IPF. This conclusion is supported by the observation that plasma ANP and cGMP levels were only significantly increased by combination therapy.

In summary, a combination therapeutic strategy targeted to enhance natriuretic peptide activity (NEPi) and prevent cGMP catabolism (PDE5i) is a potent and effective combination that prevents and reverses the fibrosis and accompanying PH in bleomycin-induced lung inflammation and injury. Moreover, natriuretic peptides represent an intrinsic cytoprotective pathway that is triggered during the pathogenesis of pulmonary fibrosis and that offsets disease progression. Therefore, this NEPi/PDE5i drug combination might be a novel therapeutic approach in IPF, which currently has little or no disease-modifying therapy, and consequently significant associated morbidity and mortality. As both components of the combination are licensed drugs, efficacy in IPF patients can be evaluated rapidly and inexpensively. This approach, therefore, shows promise to improve outcome and lower healthcare costs in this patient cohort.

## Abbreviations

αSMA: α smooth muscle actin
ANP: atrial natriuretic peptide
BNP: brain natriuretic peptide
ILD: interstitial lung disease
iNOS: inducible NO synthase
IPF: idiopathic pulmonary fibrosis
MABP: mean arterial blood pressure
NEPi: neutral endopeptidase inhibitor
NPR: natriuretic peptide receptor
PDE5i: phosphodiesterase 5 inhibitor
PH: pulmonary hypertension
RVH: right ventricular hypertrophy
RVSP: right ventricular systolic pressure
WT: wild-type

## References

[b1] Alexander SPH, Benson HE, Faccenda E, Pawson AJ, Sharman JL, Spedding M (2013). The Concise Guide to PHARMACOLOGY 2013/14: Catalytic Receptors. Br J Pharmacol.

[b2] Baliga RS, Zhao L, Madhani M, Lopez-Torondel B, Visintin C, Selwood D (2008). Synergy between natriuretic peptides and phosphodiesterase 5 inhibitors ameliorates pulmonary arterial hypertension. Am J Respir Crit Care Med.

[b3] Behnes M, Hoffmann U, Lang S, Weiss C, Ahmad-Nejad P, Neumaier M (2011). Transforming growth factor beta 1 (TGF-beta 1) in atrial fibrillation and acute congestive heart failure. Clin Res Cardiol.

[b4] Behr J, Ryu JH (2008). Pulmonary hypertension in interstitial lung disease. Eur Respir J.

[b5] Bender AT, Beavo JA (2006). Cyclic nucleotide phosphodiesterases: molecular regulation to clinical use. Pharmacol Rev.

[b6] Bianchi C, Gutkowska J, Thibault G, Garcia R, Genest J, Cantin M (1985). Radioautographic localization of 125I-atrial natriuretic factor (ANF) in rat tissues. Histochemistry.

[b7] Bjoraker JA, Ryu JH, Edwin MK, Myers JL, Tazelaar HD, Schroeder DR (1998). Prognostic significance of histopathologic subsets in idiopathic pulmonary fibrosis. Am J Respir Crit Care Med.

[b8] Border WA, Noble NA (1994). Transforming growth factor beta in tissue fibrosis. N Engl J Med.

[b9] Burgess HA, Daugherty LE, Thatcher TH, Lakatos HF, Ray DM, Redonnet M (2005). PPARgamma agonists inhibit TGF-beta induced pulmonary myofibroblast differentiation and collagen production: implications for therapy of lung fibrosis. Am J Physiol Lung Cell Mol Physiol.

[b10] Burghuber OC, Hartter E, Punzengruber C, Weissel M, Woloszczuk W (1988). Human atrial natriuretic peptide secretion in precapillary pulmonary hypertension. Clinical study in patients with COPD and interstitial fibrosis. Chest.

[b11] Campbell DJ (2003). Vasopeptidase inhibition: a double-edged sword?. Hypertension.

[b12] Condliffe R, Kiely DG, Peacock AJ, Corris PA, Gibbs JS, Vrapi F (2009). Connective tissue disease-associated pulmonary arterial hypertension in the modern treatment era. Am J Respir Crit Care Med.

[b13] Das S, Au E, Krazit ST, Pandey KN (2010). Targeted disruption of guanylyl cyclase-A/natriuretic peptide receptor-A gene provokes renal fibrosis and remodeling in null mutant mice: role of proinflammatory cytokines. Endocrinology.

[b14] Eickelberg O, Selman M (2010). Update in diffuse parenchymal lung disease 2009. Am J Respir Crit Care Med.

[b15] Farkas L, Gauldie J, Voelkel NF, Kolb M (2011). Pulmonary hypertension and idiopathic pulmonary fibrosis: a tale of angiogenesis, apoptosis, and growth factors. Am J Respir Cell Mol Biol.

[b16] Gasse P, Mary C, Guenon I, Noulin N, Charron S, Schnyder-Candrian S (2007). IL-1R1/MyD88 signaling and the inflammasome are essential in pulmonary inflammation and fibrosis in mice. J Clin Invest.

[b17] Gutkowska J, Cantin M, Genest J, Sirois P (1987). Release of immunoreactive atrial natriuretic factor from the isolated perfused rat lung. FEBS Lett.

[b18] Hulks G, Jardine AG, Connell JM, Thomson NC (1990). Effect of atrial natriuretic factor on bronchomotor tone in the normal human airway. Clin Sci (Lond).

[b19] Kapoun AM, Liang F, O'Young G, Damm DL, Quon D, White RT (2004). B-type natriuretic peptide exerts broad functional opposition to transforming growth factor-beta in primary human cardiac fibroblasts: fibrosis, myofibroblast conversion, proliferation, and inflammation. Circ Res.

[b20] Kenny AJ, Stephenson SL (1988). Role of endopeptidase-24.11 in the inactivation of atrial natriuretic peptide. FEBS Lett.

[b21] Kilkenny C, Browne W, Cuthill IC, Emerson M, Altman DG (2010). Animal research: reporting *in vivo* experiments: the ARRIVE guidelines. Br J Pharmacol.

[b22] Klinger JR, Thaker S, Houtchens J, Preston IR, Hill NS, Farber HW (2006). Pulmonary hemodynamic responses to brain natriuretic peptide and sildenafil in patients with pulmonary arterial hypertension. Chest.

[b23] Knowles JW, Esposito G, Mao L, Hagaman JR, Fox JE, Smithies O (2001). Pressure-independent enhancement of cardiac hypertrophy in natriuretic peptide receptor A-deficient mice. J Clin Invest.

[b24] Koga H, Hagiwara S, Shingu C, Matsumoto S, Yokoi I, Noguchi T (2010). Human atrial natriuretic peptide ameliorates LPS-induced acute lung injury in rats. Lung.

[b25] Kolodsick JE, Toews GB, Jakubzick C, Hogaboam C, Moore TA, McKenzie A (2004). Protection from fluorescein isothiocyanate-induced fibrosis in IL-13-deficient, but not IL-4-deficient, mice results from impaired collagen synthesis by fibroblasts. J Immunol.

[b26] Ladetzki-Baehs K, Keller M, Kiemer AK, Koch E, Zahler S, Wendel A (2007). Atrial natriuretic peptide, a regulator of nuclear factor-{kappa}B activation in vivo. Endocrinology.

[b27] Lee CY, Burnett JC (2007). Natriuretic peptides and therapeutic applications. Heart Fail Rev.

[b28] Lettieri CJ, Nathan SD, Barnett SD, Ahmad S, Shorr AF (2006). Prevalence and outcomes of pulmonary arterial hypertension in advanced idiopathic pulmonary fibrosis. Chest.

[b29] Leuchte HH, Neurohr C, Baumgartner R, Holzapfel M, Giehrl W, Vogeser M (2004). Brain natriuretic peptide and exercise capacity in lung fibrosis and pulmonary hypertension. Am J Respir Crit Care Med.

[b30] Ley B, Collard HR, King TE (2011). Clinical course and prediction of survival in idiopathic pulmonary fibrosis. Am J Respir Crit Care Med.

[b31] Li P, Wang D, Lucas J, Oparil S, Xing D, Cao X (2008). Atrial natriuretic peptide inhibits transforming growth factor beta-induced Smad signaling and myofibroblast transformation in mouse cardiac fibroblasts. Circ Res.

[b32] Lo CS, Chen ZH, Hsieh TJ, Shin SJ (2008). Atrial natriuretic peptide attenuates high glucose-activated transforming growth factor-beta, Smad and collagen synthesis in renal proximal tubular cells. J Cell Biochem.

[b33] Maher TM, Wells AU, Laurent GJ (2007). Idiopathic pulmonary fibrosis: multiple causes and multiple mechanisms?. Eur Respir J.

[b34] Martinez FJ, Safrin S, Weycker D, Starko KM, Bradford WZ, King TE (2005). The clinical course of patients with idiopathic pulmonary fibrosis. Ann Intern Med.

[b35] McGrath J, Drummond G, McLachlan E, Kilkenny C, Wainwright C (2010). Guidelines for reporting experiments involving animals: the ARRIVE guidelines. Br J Pharmacol.

[b36] Mejia M, Carrillo G, Rojas-Serrano J, Estrada A, Suarez T, Alonso D (2009). Idiopathic pulmonary fibrosis and emphysema: decreased survival associated with severe pulmonary arterial hypertension. Chest.

[b37] Moriyama N, Taniguchi M, Miyano K, Miyoshi M, Watanabe T (2006). ANP inhibits LPS-induced stimulation of rat microglial cells by suppressing NF-kappaB and AP-1 activations. Biochem Biophys Res Commun.

[b38] Naura AS, Zerfaoui M, Kim H, Abd Elmageed ZY, Rodriguez PC, Hans CP (2010). Requirement for inducible nitric oxide synthase in chronic allergen exposure-induced pulmonary fibrosis but not inflammation. J Immunol.

[b39] Nishikimi T, Inaba-Iemura C, Ishimura K, Tadokoro K, Koshikawa S, Ishikawa K (2009). Natriuretic peptide/natriuretic peptide receptor-A (NPR-A) system has inhibitory effects in renal fibrosis in mice. Regul Pept.

[b40] Noble PW, Albera C, Bradford WZ, Costabel U, Glassberg MK, Kardatzke D (2011). Pirfenidone in patients with idiopathic pulmonary fibrosis (CAPACITY): two randomised trials. Lancet.

[b41] Perreault T, Gutkowska J (1995). Role of atrial natriuretic factor in lung physiology and pathology. Am J Respir Crit Care Med.

[b42] Piguet PF, Ribaux C, Karpuz V, Grau GE, Kapanci Y (1993). Expression and localization of tumor necrosis factor-alpha and its mRNA in idiopathic pulmonary fibrosis. Am J Pathol.

[b43] Potter LR, Yoder AR, Flora DR, Antos LK, Dickey DM (2009). Natriuretic peptides: their structures, receptors, physiologic functions and therapeutic applications. Handb Exp Pharmacol.

[b44] Preston IR, Hill NS, Gambardella LS, Warburton RR, Klinger JR (2004). Synergistic effects of ANP and sildenafil on cGMP levels and amelioration of acute hypoxic pulmonary hypertension. Exp Biol Med (Maywood).

[b45] Sakamoto M, Nakao K, Morii N, Sugawara A, Yamada T, Itoh H (1986). The lung as a possible target organ for atrial natriuretic polypeptide secreted from the heart. Biochem Biophys Res Commun.

[b46] Saleh D, Barnes PJ, Giaid A (1997). Increased production of the potent oxidant peroxynitrite in the lungs of patients with idiopathic pulmonary fibrosis. Am J Respir Crit Care Med.

[b47] Scotton CJ, Chambers RC (2007). Molecular targets in pulmonary fibrosis: the myofibroblast in focus. Chest.

[b48] Scotton CJ, Chambers RC (2010). Bleomycin revisited: towards a more representative model of IPF?. Am J Physiol Lung Cell Mol Physiol.

[b49] Scotton CJ, Krupiczojc MA, Konigshoff M, Mercer PF, Lee YC, Kaminski N (2009). Increased local expression of coagulation factor X contributes to the fibrotic response in human and murine lung injury. J Clin Invest.

[b50] Soeki T, Kishimoto I, Okumura H, Tokudome T, Horio T, Mori K (2005). C-type natriuretic peptide, a novel antifibrotic and antihypertrophic agent, prevents cardiac remodeling after myocardial infarction. J Am Coll Cardiol.

[b51] Soleilhac JM, Lucas E, Beaumont A, Turcaud S, Michel JB, Ficheux D (1992). A 94-kDa protein, identified as neutral endopeptidase-24.11, can inactivate atrial natriuretic peptide in the vascular endothelium. Mol Pharmacol.

[b52] Stasch JP, Knorr A, Wegner M, Hirth-Dietrich C (1995). Prolonged inhibition of neutral endopeptidase 24.11 by sinorphan in stroke-prone spontaneously hypertensive rats. Hypertens Res.

[b53] Sun M, Fink PJ (2007). A new class of reverse signaling costimulators belongs to the TNF family. J Immunol.

[b54] Tamura N, Ogawa Y, Chusho H, Nakamura K, Nakao K, Suda M (2000). Cardiac fibrosis in mice lacking brain natriuretic peptide. Proc Natl Acad Sci U S A.

[b55] Thavarajah K, Wu P, Rhew EJ, Yeldandi AK, Kamp DW (2009). Pulmonary complications of tumor necrosis factor-targeted therapy. Respir Med.

[b56] Toshimori H, Nakazato M, Toshimori K, Asai J, Matsukura S, Oura C (1988). Distribution of atrial natriuretic polypeptide (ANP)-containing cells in the rat heart and pulmonary vein. Immunohistochemical study and radioimmunoassay. Cell Tissue Res.

[b57] Willis BC, Borok Z (2007). TGF-beta-induced EMT: mechanisms and implications for fibrotic lung disease. Am J Physiol Lung Cell Mol Physiol.

[b58] Wynn TA (2003). IL-13 effector functions. Annu Rev Immunol.

[b59] Zhang Y, Lee TC, Guillemin B, Yu MC, Rom WN (1993). Enhanced IL-1 beta and tumor necrosis factor-alpha release and messenger RNA expression in macrophages from idiopathic pulmonary fibrosis or after asbestos exposure. J Immunol.

[b60] Zhao L, Mason NA, Morrell NW, Kojonazarov B, Sadykov A, Maripov A (2001). Sildenafil inhibits hypoxia-induced pulmonary hypertension. Circulation.

[b61] Zhao L, Mason NA, Strange JW, Walker H, Wilkins MR (2003). Beneficial effects of phosphodiesterase 5 inhibition in pulmonary hypertension are influenced by natriuretic Peptide activity. Circulation.

